# Biometrics Analysis and Evaluation on Korean *Makgeolli* Using Brainwaves and Taste Biological Sensor System

**DOI:** 10.1155/2015/918631

**Published:** 2015-07-13

**Authors:** Yong-Sung Kim, Yong-Suk Kim

**Affiliations:** ^1^Division of Computer Science & Engineering, Chonbuk National University, Jeonju 561-756, Republic of Korea; ^2^Department of Food Science and Technology, Chonbuk National University, Jeonju 561-756, Republic of Korea

## Abstract

There are several methods available in measuring food taste. The sensory evaluation, for instance, is a typical method for panels to test of taste and recognize smell with their nose by measuring the degree of taste characteristic, intensity, and pleasure. There are many issues entailed in the traditional sensory evaluation method such as forming a panel and evaluation cost; moreover, it is only localized in particular areas. Accordingly, this paper aimed to select food in one particular area, and compare and review the content between sensory evaluations using a taste biological sensor, as well as presenting an analysis of brainwaves using EEG and finally a proposal of a new method for sensory evaluation. In this paper, the researchers have conducted a sensory evaluation whereas a maximum of nine points were accumulated by purchasing eight types of rice wine. These eight types of *Makgeolli* were generalized by generating multidimensional data with the use of TS-5000z, thus learning mapping points and scaling them. The contribution of this paper, therefore, is to overcome the disadvantages of the sensory evaluation with the usage of the suggested taste biological sensor system.

## 1. Introduction

Recently, many strategic plans have been proposed to globalize Korean food. Yet, there were various issues to take Korean food globally in accordance with such proposals. Not only has the degree of taste, characteristic, intensity, and pleasure formed the backbone of taste for the actual food, but also taste differs depending on the country and regional environment. Because a lot of time and cost are involved in researching and analyzing such conditions, new alternative plan is required to globalize Korean food [1].

In this paper, in order to overcome human limit and economic feasibility with regard to sensory evaluation, comparing and analyzing evaluation results between the taste biological sensor system (*TS-5000z)* and sensory evaluation of panels with physicochemical analysis have been conducted.

Subsequently, it continued to analyze the results of brainwaves of panels to resulting materials of* TS-5000z* to draw out standardized results of characteristics and intensity to a certain degree that forms the backbone of taste.

Standardization and taste measurement are needed in order to globalize alcoholic beverages making it commercially available everywhere. In relation to the case of European wines, enhancing the product's value and improved productivity is regarded through its taste specialization by its brand.* Makgeolli*, for thousands of years, is one of the most marketable traditional alcoholic beverages in Korea.

There were especially made adjective-expressions for sensory evaluation that are well developed in the Korean language, hence, the existence of numerous adjective terms to express the taste of* Makgeolli*. According to the previous pilot research, with regard to the mutual similarities of the pairs of terms, we can reach the realization of the hardship of formulating complete sets with small number of adjectives that can generally be used to express all kinds of Korean* Makgeolli.*

*Makgeolli* is an alcoholic drink made with rice, wheat flour, barley, corn, and sweet potato as major fermenting materials and thereafter fermented with* Koji* or* Nuruk* [1, 2]. The physicochemical and taste qualities of this drink depend on the making materials, heating methods, and* Nuruk, *as well as the produced area [3]. In order for this drink to maintain its quality characteristics,* Makgeolli* has to be heated at 62–65°C for 30 minutes, preventing the drink from being altered for a long period of time to be able to keep its flavor and balanced taste. Sterilized* Makgeolli*, however, has weaknesses such as death of useful microbes, heated flavor, and a feeling of refreshment [3, 4].

This paper, therefore, concentrates on the elicitation of regional properties found in* Makgeolli *through the taste correlation between the electroencephalographic data of sensual test and taste biological sensor data.

Accordingly, by analyzing correlation of physicochemical analysis and sensory evaluation of* Makgeolli* with* TS-5000z* analysis results and by comparing analyzed results of brainwaves of panels based on such findings, it showed that it has the capability to replace the sensory evaluation as a scale if complementary scale is proposed for the analyzed results of* TS-5000z*.

## 2. Related Works

### 2.1. Taste-Adjectives

The taste is often described as a sensory reaction that is caused by the chemical stimulus. In order to recognize the taste, materials are firstly dissolved in the saliva that stimulates gustatory cells, hence signaling the cerebrum. Hereafter, the activity takes about a minute to taste. After eating the food, the taste lingers for 30 seconds.

**Figure 1:**
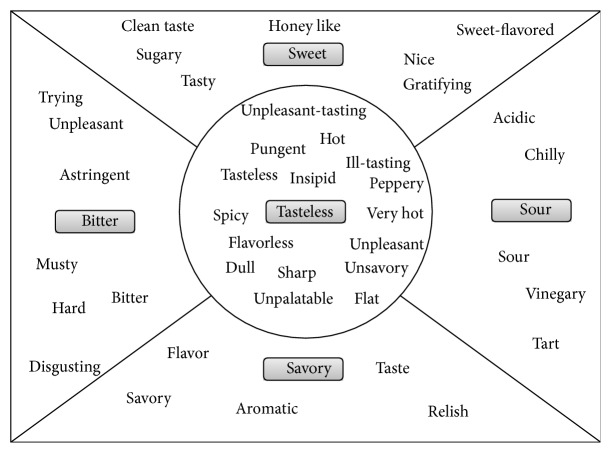
Type of taste information.

Scale describing and introducing a particular product in qualitative and quantitative measures is called a scale method. Four basic tastes in Figure 1 are the baseline for dimensions in accordance with “imputed characteristic to a certain taste [5, 6].”

Here, the closer the concept of a particular adjective is to a traditional taste, the closer that concept gets to the baseline. However, it is difficult to argue this type of ordering as it is affiliated with dimensions in accordance with imputed characteristic to a certain taste.

Since quantitative description is defined with characteristics and intensity of appearance, smell, taste, and physical properties of a particular product, it is typical to understand taste in terms of physiological base and taste-adjectives formed in its semantics.

There is a point to consider when classifying the properties of* Makgeolli*: although a certain taste of its property is generally classified as good, it can still be recognized as unpleasant if it shows more than the required specific concentration.

### 2.2. Clustering Algorithm

Taste evaluation scale development using adjective pairs is extremely small and although there have been cases of using food taste as evaluation scale, it proved to be unsuccessful. In order to develop adjective scale that is to be used in qualitative sensory evaluation of actual taste, it is necessary to use correlation of adjectives to analyze factor analysis and congregation analysis and congregate adjectives expressing positive taste, negative taste, food texture, and heat level. In general, congregation is defined as follows.

Let us say that the sample *S* = {*x*_1_, *x*_2_, …, *x*_*n*_} is given, which satisfies the below conditional equation, a subset of *C* = {*S*_1_, *S*_2_, …, *S*_*c*_}:

(1) $$\begin{array}{c} S_{i}\neq ⌀,\;\left(i=1,\ldots ,c\right)\\ S_{i}\cap S_{j}=⌀,\;\left(i,j=1,\ldots ,c,\;\;i\neq j\right)\\ \overset{c}{\underset{i=1}{⋃}}=S. \end{array}$$

Affiliated variables are defined in expression 2 and congregationl number in expression 3. Each congregation uses variables that show the degree of affiliation. Affiliated variable* m*_*ij*_ shows the degree of affiliation of *x*_*i*_ toward *x*_*j*_. Affiliated variables should satisfy the following conditional equation:

(2) $$\begin{array}{c} 0\leq m_{ij}\leq 1,\\ \overset{k}{\underset{j=1}{\sum }}m_{ij}=1,\\ \overset{N}{\underset{i=1}{\sum }}m_{ij}<N. \end{array}$$

Stirling number may be utilized to count different types of congregations. Stirling number is a conditional number from dividing objects of *N* onto *K* group. Therefore, Stirling number can be defined cyclic expression as follows:

(3) SN,N=1, k=1  or  Notherwise,SN,k=SN−1,k−1+kSN−1,k.

In this paper, termite colony algorithm is used to congregate adjectives used to describe* Makgeolli*. Termite colony algorithm is based on the probability value of termite behavior. The aim is to find a suitable initial congregation required for *k*-means congregation using termite colony algorithm. Through termite search, estimating sample density within congregation is possible, and this estimated initial set suitable for density influences congregational functions (Algorithm 1).

### 2.3. Samples and Analysis Biometrics

With the purpose of experimenting, 12 commercialized* Makgeolli* samples (unsterilized = 5, sterilized = 7) produced in South Korea were purchased and maintained at 4°C refrigerator. The five unsterilized commercial* Makgeolli* samples were categorized as US1, US2, US3, US4, and US5. On the other hand, seven sterilized samples were categorized as S1, S2, S3, S4, S5, S6, and S7 [7].

The physicochemical qualities of* Makgeolli* samples such as pH, aminotype nitrogen, titratable acidity, and soluble solid contents were tested [8, 9]. In the case of sensory testing, graduate and undergraduate students (*n* = 25) from the Department of Food Science and Technology in Chonbuk National University were introduced with the objective and methodology prior to the test. The samples were tested using a 9-point hedonic scale ranging from “really like” (scale 9) to “really dislike” (scale 1). Properties like turbidity, color, flavor, sweetness, sourness, bitterness, thickness, cooling sensation, and balance were evaluated for the sensory test [9].

The data was treated using the statistical analysis system (SAS, 1998) package software for the analysis of variance and Duncan's test. All analyses were conducted in triplicate except for the sensory test that was measured by 25 students. The statistical significance was established at *P* < 0.05.

### 2.4. Brainwaves

**Figure 2:**
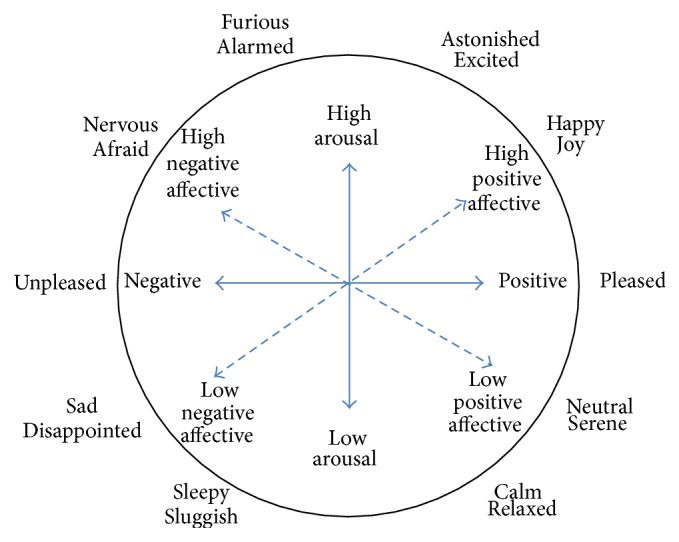
Valence-arousal emotional model.

Brainwave research with regard to human emotional change has been relatively developed. This research used a dimensional approach, derived from the cognitive theories, which determines a person's state of mind in terms of dimension. The common dimensions are determined through two types: the valence of emotion, reflecting either positive or negative emotions, and the arousal level of emotion, reflecting either active or passive emotion [6, 8].  Figure 2 is a 2D valence-arousal emotional model. The model shows more diversified and generalized expressions compared to discrete emotion approaches.

EEG (electroencephalogram) is a brain activity classified with brainwaves that emit electric flow which is generated when signals are sent out into the cerebral nerves through the nervous system. When measuring brainwaves, it is possible to obtain an exceptionally complicated type of analogue waveform as seen in the valence-arousal emotional model. It is considered as the raw data of EEG. The raw data, afterwards, were transformed into digital data [10]. The digital data, subsequently, were transformed into a power spectrum, whereas it was also utilized for data analysis.

Power spectral analysis is a mathematical approach employed in order to quantify EEG. It does not, however, provide a biophysical model of the EEG generation. The purpose of the analysis is for the decomposition of signals into its constituting frequency components (e.g., EEG). FFT (fast Fourier transform), on the other hand, is known as a widely used method in acquiring data such as the EEG spectrum [11–13].

FFT algorithm describes signals as linear superposition of Sines and Cosines characterized by their frequency in the equation as seen below expression 4. Consider

(4) $$\begin{array}{c} x\left(t\right)=\overset{+\infty }{\underset{-\infty }{\int }}X\left(f\right)e^{i2\pi ft}df. \end{array}$$

The power spectrum, also known as power density spectrum, demonstrates the distribution of power or variance over the frequency components of a signal. It is also known as a Fourier transform of the autocorrelation function.

The degree of the brain activity causes the shape of brainwaves to react differently. If the brain works more actively, it produces a wider frequency bandwidth of brainwaves as seen in Table 1.

One of the most used methods in the analysis of brainwaves is the power spectrum analysis [12, 13]. This study, hence, mainly utilized the power spectrum analysis since it is a remarkably adequate method in terms of the time series frequency analysis of the raw data.

## 3. Experimental Results and Discussion

### 3.1. Representative Taste-Adjective

**Figure 3:**
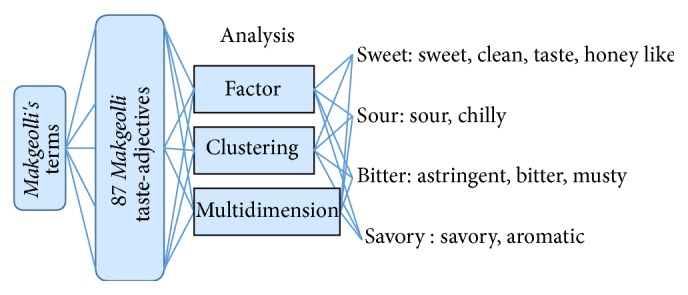
Four catalog of* Makgeolli* taste-adjectives.

Before evaluating eight different types of* Makgeolli* purchased locally through sensory evaluation and* TS-5000z* taste sensor, there was little preliminary work that needs to be done. First was the selection of* Makgeolli* that sells the most locally. Secondly, get 20 students to sort out 87 taste-adjectives that were used on Internet and in Korean dictionary. Thirdly, out of the 87 selected taste-adjectives, 45 of them were sorted out and were placed into final four categories. Lastly, through factor analysis and congregation algorithm as well as multidimensional analysis, we extracted 12 taste-adjectives for* Makgeolli*. The processed result is shown in Figure 3.

The outcome of the experiment leads into four representative* Makgeolli-*adjectives describing taste: sweet, sour, bitter, and savory.

As shown above, among various* Makgeolli* types in adjective dimension, finding the* Makgeolli* that is preferred by foreigners should help narrowing down the taste dimension of* Makgeolli* from adjective dimension for exporting the product globally.

### 3.2. Physicochemical Characteristics of* Makgeolli*

**Figure 4:**
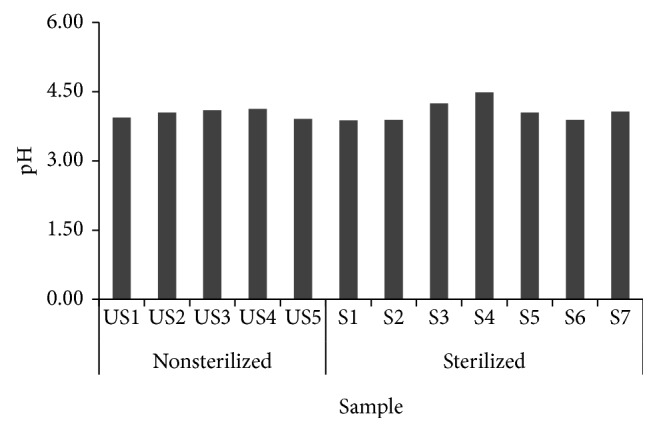
pH of 12* Makgeolli* samples produced in South Korea.

The pH level of* Makgeolli*, as an alcoholic drink, is a significant factor for its preservation and fermentation process. The pH of S1* Makgeolli *sample, as seen in Figure 4, had the lowest level with 3.88 while that of S4 sample had the highest pH with 4.49.

**Figure 5:**
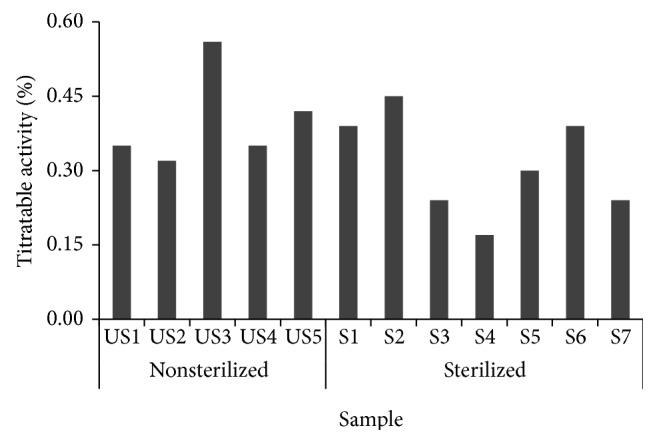
Titratable acidity of 12* Makgeolli* samples produced in South Korea.

Titratable acidity, on the other hand, serves as a vital indicator influencing the taste and flavor of a drink. The lactic acid bacteria and yeast found in* Makgeolli *produce organic acid that can be increased within the process of fermentation [14]. The result in Figure 5 shows that the titratable acid content of the S4 sample (sterilized) had the lowest percentage with 0.17%. Meanwhile, the US3 which got 0.56 percent had the highest percentage within the samples.

**Figure 6:**
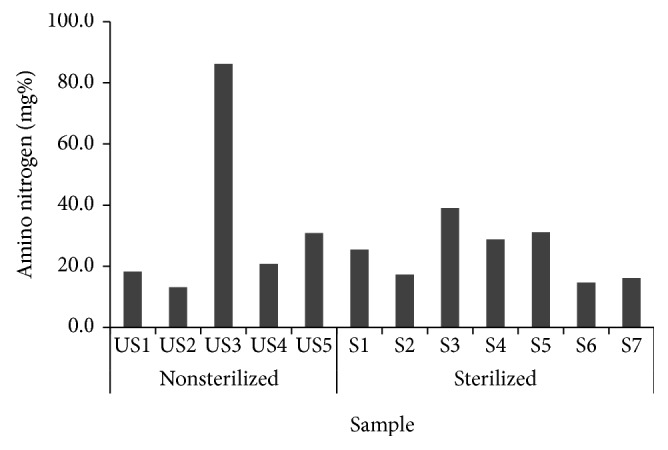
Aminotype nitrogen contents of 12* Makgeolli* samples produced in South Korea.

The aminotype nitrogen contents were illustrated in Figure 6 resulting with US3 (sterilized) with 86.19 mg% being the highest among the samples while the other samples ranged from 13.08 to 31.06 mg%.

**Figure 7:**
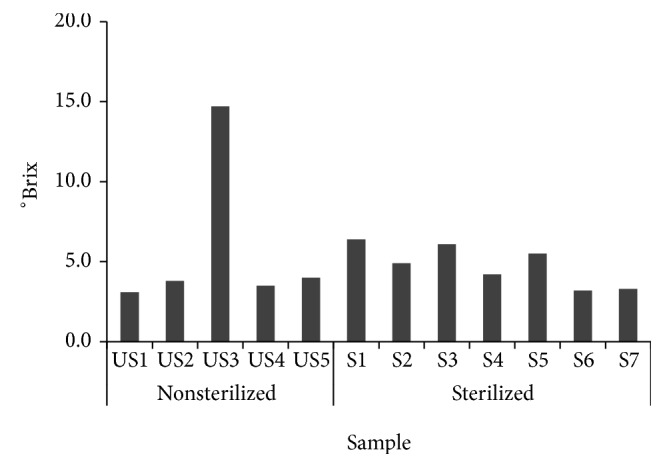
Soluble solid contents of 12* Makgeolli* samples produced in South Korea.

In Figure 7, the soluble solid contents of US3 (unsterilized), S1, and S3 (sterilized) obtained 14.7, 6.4, and 6.1°Brix.

### 3.3. Sensory Test on Domestic* Makgeolli *Samples Using Taste Biological Sensor

Taste biological sensor, an electronic equipment, has a similar structure as the taste bud of a human tongue and detects taste ingredient, which is sent to a computer in corresponding electrical signal which compiles data.

**Figure 8:**
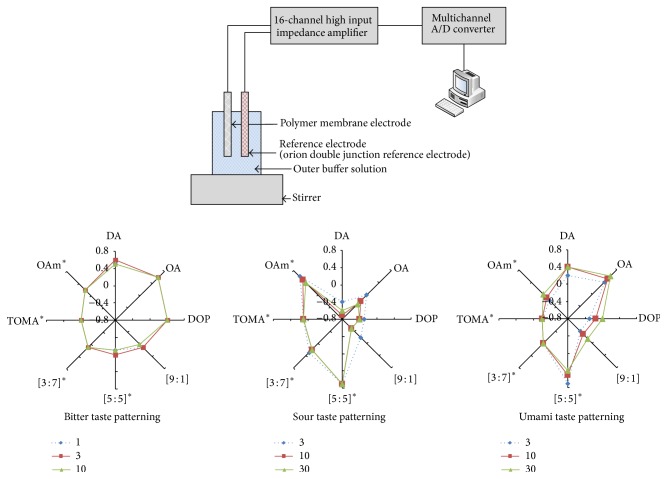
Taste biological sensor (*TS-5000z) *and multidimensional data.

The taste was analyzed using* TS-5000z*, a taste biological sensor from Japan. An available taste biological sensor system found in Daesang Research Center from Incheon, Korea, was used for equipment testing as seen in Figure 8. The system consists of five taste biological sensors, namely, sourness, umami, bitterness, saltiness, and astringency. There were also three aftertaste sensors classified as bitterness, astringency, and umami [15].

**Figure 9:**
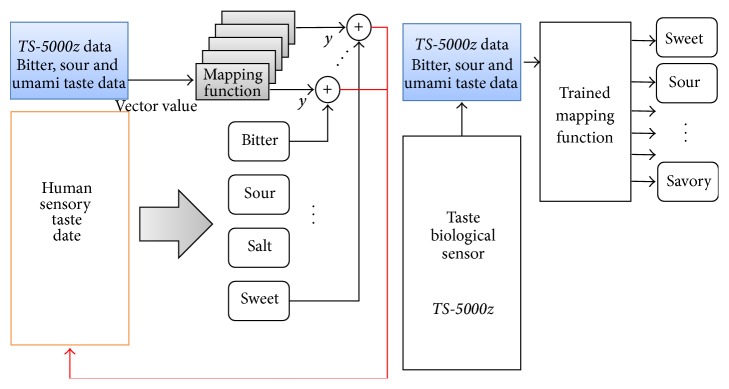
Structure of learning process.

The method for converting multidimensional data measured by* TS-5000z* to a taste form recognized by human is important. In other words, the required implementation of mapping function of four  (4) adjective scales of* Makgeolli* abstracted and measured from* TS-5000z*, and in order to verify these brainwaves of panels are used as in Figure 9.

In this paper, eight different types of* Makgeolli* have been purchased that are currently sold locally to analyze physicochemical and microbiological properties. Afterwards, a 9-point scale sensory evaluation is conducted, and using eight types of* Makgeolli* evaluated by these panels, multidimensional data has been created from* TS-5000z* and studied through mapping function.

Using the statistical analysis system (SAS, 1998) package software, the data was analyzed for the analysis of variance and Duncan's test. All analyses were carried out in triplicate with the exception of the sensory evaluation that was measured by 12 determinations. The significance was established at *P* < 0.05.

The results on the 12 domestic* Makgeolli* samples using the taste biological sensor system were shown in Table 2. The samples US2 (6.00 ± 2.11), US4 (5.80 ± 1.99), S1 (5.50 ± 1.43), and S2 (5.50 ± 1.27) were significantly high in balance. Moreover, the samples S6, S2, and S1 (sterilized) scored higher with 6.40, 6.00, and 5.70 compared to other samples. The significant differences were not observed between sterilized and unsterilized samples in balance scores of sensory test within the use of this system.

### 3.4. Taste Biological Sensor Analyses on* Makgeolli*

**Figure 10:**
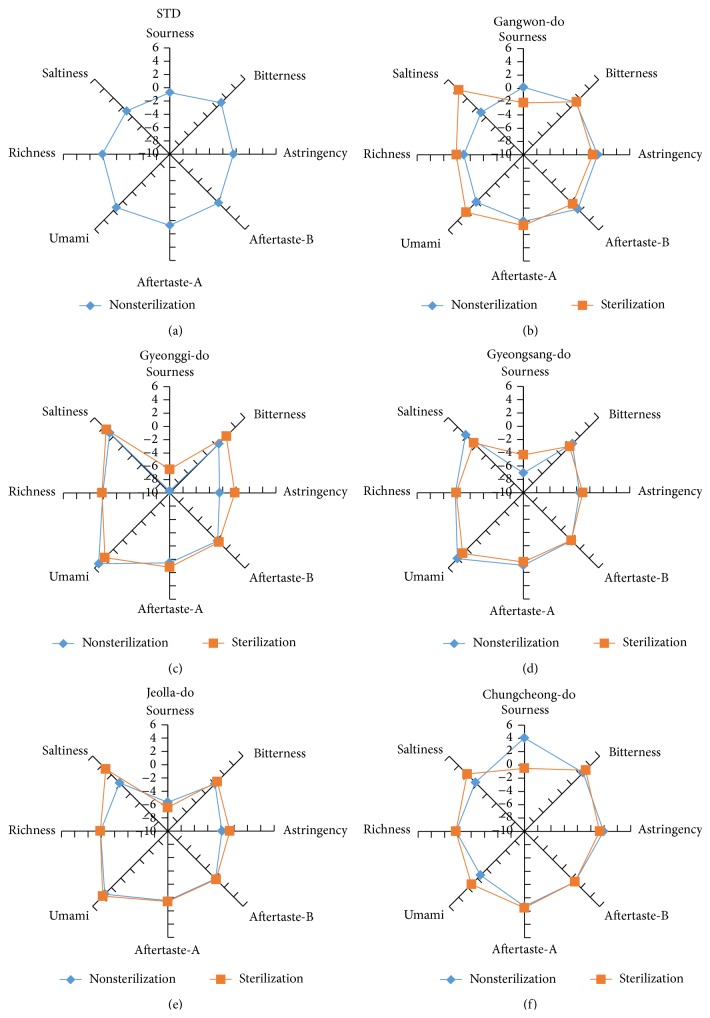
Taste results by taste biological sensor on 12* Makgeolli* samples produced in South Korea.

The taste biological sensor was compared to a standard one. The standard sample which showed middle score in the sensory test was the standard sample CD1 (unsterilized). According to taste biological sensor test results, as shown in Figure 10, the sourness level showed significant distinction between WM1 (7.69) and BS1 (−23.84). Meanwhile, the sourness found in an unsterilized* Makgeolli *sample was generally higher than that in sterilized ones.

The bitterness found in the samples CJ1 (5.59), SJ1 (5.56), WR2 (5.47), WR1 (5.20), CW1 (5.13), and SD1 (5.03) was high. Umami and richness, on the other hand, found in the samples GS5, WR1, PG1, GS1, and SJ1 were higher than the other samples. The SD1 sample showed the highest score in umami with 6.65 and 0.83 in richness according to the taste biological sensor.

### 3.5. Correlation

The correlation analysis between the analytical value of physicochemical characteristics and sensory testing of Korean* Makgeolli *samplesusing the taste biological sensor was conducted.

The pH and titratable acidity (physicochemical characteristics), as shown in Table 3, showed high reverse correlation. The correlation analysis between physicochemical characteristics and sensory testing of Korean* Makgeolli *using the taste biological sensor showed that “titratable acidity” and “sourness, cooling sensation, and balance” have high correlation.

In addition, the criteria “sweetness-bitterness,” “sourness-bitterness, thickness, cooling sensation, and balance,” “bitterness-thickness, cooling sensation, and balance,” “thickness- cooling sensation and balance,” and “cooling sensation-balance” showed a strong correlation as shown in Table 3. Lastly, items between physicochemical characteristic analysis and taste biological sensor showed a strong correlation score.

### 3.6. Brainwaves Detection

The EEG used in this research was produced by the American Neuroscience. The mindset of the said EEG has a series of advantages. Firstly, EEG can distinguish the state of brain; in other words, it can differentiate good state from bad state. Secondly, because EEG can analyze brainwaves in real-time, this paper analyzed brainwaves in a five-minute lapse after tasting* Makgeolli* of the panels. Lastly, because EEG outputs brainwaves in power spectrum form this has been digitized for utilization [16].

**Figure 11:**
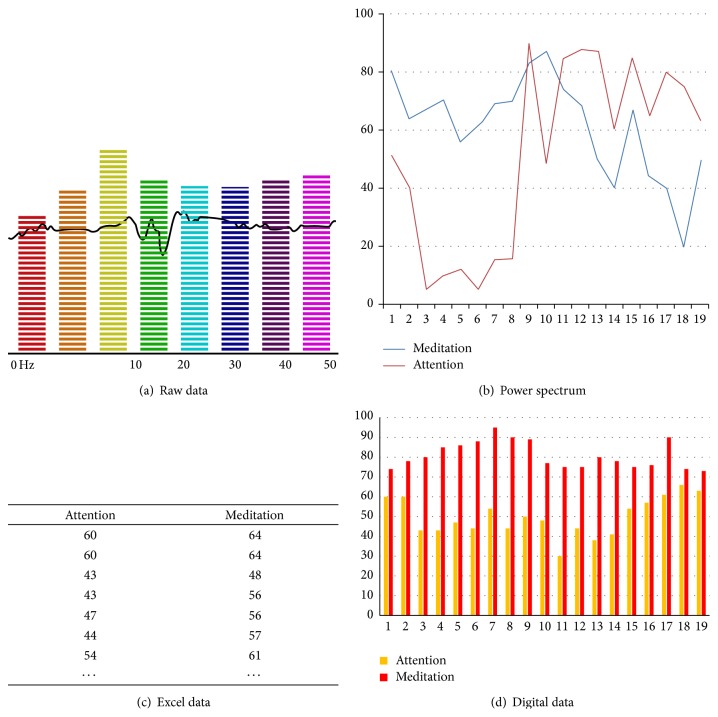
Digital transformation process.

Analog data obtained from raw data of the panels' brainwaves have been converted from power spectrum form to digital data through FFT, which is then visualized through excel data as seen in Figure 11 [16, 17].

**Figure 12:**
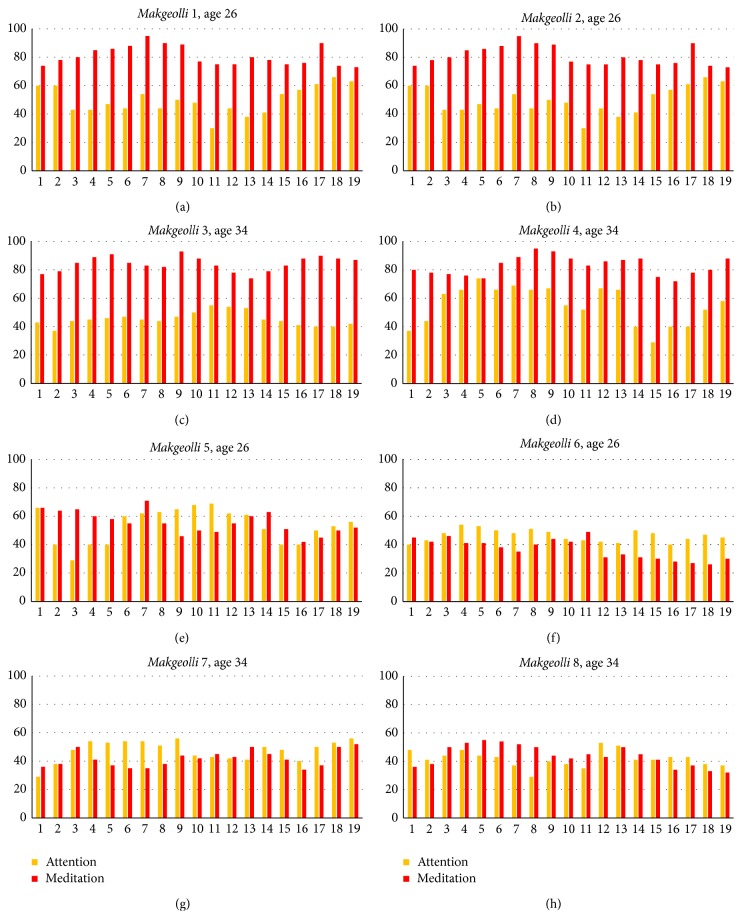
Analysis results of brainwaves.

Power spectrum analysis shows change in the process from good state to bad state using raw data that is generated from time series, which is evaluated by the tasting of panels of the eight* Makgeolli *samples. Since numerical analysis of brainwaves with regard to selecting the* Makgeolli* changes over time, after a five-minute lapse from tasting* Makgeolli*, the intensity of taste is measured 10 times in one minute. In other words, within just two minutes, measuring was done.  Figure 12 shows the tasting results of four panels who have consumed eight* Makgeolli* samples in two minutes.

As shown in Figure 12,* Makgeolli* samples 1, 2, 3, and 4 show good state whereas samples 7 and 8 show bad state, and 5 and 6 show the in-between state. This experiment result was almost in match with experiment result of* TS-5000z*.

## 4. Conclusion

It may be said that, strategically, coming up with a scale that can evaluate food's taste characteristic, intensity, and pleasure is one way to globalize Korean food. In general, representative method that evaluates taste is called sensory evaluation, where human recognizes food taste using the actual tongue. This however, in realistic sense, is used in only limited cases due to issues with forming panels and cost incurred in order to conduct sensory evaluation.

Accordingly, in this paper, artificial tongue from electronic equipment, in other words, suggests the method of utilizing taste biological sensor system in place of sensory evaluation. Firstly, it had chosen* Makgeolli* samples that are sold locally and congregated taste-adjectives used to describe chosen* Makgeolli* by applying it through termite colony theory.

Afterwards, the results of physics and chemical characteristic and sensory evaluation of* Makgeolli* have been compared with the measured result from the taste biological sensor and scaled abstract function in order to suggest a generalized scale.

Therefore, the degree of contribution this paper made was from suggesting a new paradigm that may replace sensory evaluation with taste biological sensor and a method that overcomes various shortcomings of sensory evaluation. Moreover, in order to obtain precise and exact result, it had provided a standardized scale of taste biological sensor by comparing tasting results of* Makgeolli*.

This study is a pioneer in contributing through the investigation between the correlation analysis of brainwaves in accordance with taste biological sensor evaluation and electroencephalographic data. For future researches, there would be an expansion and comparison in a selection of liquors, especially beer and wine, including the smell and color to statistically improve the taste biological sensor system. A foundation is established for the application of the taste biological sensor system in a specific area as an alternative to sensory evaluation.

## Figures and Tables

**Algorithm 1:**
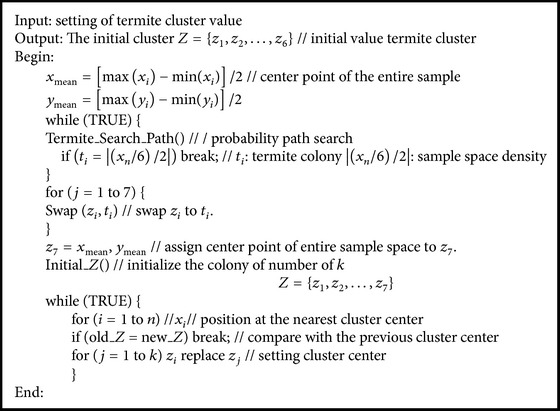
TCA *K*-mean's algorithm.

**Table 1:** Type and feature of brainwaves.

Type	Frequency	Normality
*δ* (delta)	0.5~4 Hz	Hypnoidal
*θ* (theta)	4~7 Hz	Slow wave sleep
*α* (alpha)	8~12 Hz	Stable wave
Mid-*β* (midbeta)	16~20 Hz	Concentrate stable wave
*β* (beta)	21~30 Hz	Action stress wave
*γ* (gamma)	30~50 Hz	Arousal and excitement

**Table 2:** Sensory test of 12 *Makgeolli* samples produced in South Korea using taste biological sensor.

Samples	Turbidity	Color	Flavor	Sweetness	Sourness	Bitterness	Thickness	Cooling sensation	Balance
US1	5.90 ± 1.10^abc^	6.00 ± 0.82^bcd^	5.50 ± 1.08^b^	4.70 ± 1.25^abc^	5.20 ± 1.69^ab^	5.60 ± 1.51^a^	5.10 ± 0.74^a^	6.10 ± 1.10^ab^	5.10 ± 1.10^abc^
US2	6.50 ± 1.96^a^	7.60 ± 1.43^a^	7.10 ± 1.20^a^	5.80 ± 1.93^ab^	5.80 ± 1.69^a^	5.70 ± 1.77^a^	5.00 ± 2.31^ab^	5.40 ± 1.71^abcd^	6.00 ± 2.11^a^
US3	4.30 ± 1.49^d^	3.70 ± 1.83^f^	4.90 ± 1.79^b^	3.40 ± 1.58^c^	3.00 ± 1.56^c^	3.10 ± 1.29^c^	4.50 ± 2.46^ab^	3.20 ± 2.10^ef^	3.70 ± 1.34^cde^
US4	6.60 ± 2.32^a^	7.50 ± 1.08^a^	7.10 ± 0.88^a^	6.10 ± 2.02^a^	5.60 ± 2.50^a^	5.40 ± 2.37^a^	4.50 ± 2.01^ab^	5.20 ± 2.25^abcd^	5.80 ± 1.99^ab^
US5	6.10 ± 1.37^ab^	7.00 ± 1.41^ab^	2.80 ± 1.87^c^	3.10 ± 1.66^c^	3.20 ± 2.15^bc^	3.20 ± 2.10^bc^	3.20 ± 2.10^b^	4.10 ± 2.60^cdef^	3.10 ± 1.97^e^

S1	5.20 ± 0.92^abcd^	5.20 ± 1.14^cde^	5.90 ± 1.10^ab^	5.80 ± 1.23^ab^	5.10 ± 2.13^abc^	5.00 ± 1.49^ab^	5.10 ± 1.60^a^	5.70 ± 1.64^abc^	5.50 ± 1.43^ab^
S2	4.50 ± 0.85^cd^	5.10 ± 0.99^de^	5.90 ± 1.52^ab^	5.90 ± 0.88^ab^	5.40 ± 1.84^a^	5.60 ± 1.65^a^	5.10 ± 1.45^a^	6.00 ± 1.76^ab^	5.50 ± 1.27^ab^
S3	6.20 ± 1.55^ab^	6.50 ± 1.43^abc^	5.70 ± 1.64^ab^	5.50 ± 1.84^ab^	4.40 ± 1.84^abc^	4.70 ± 2.16^abc^	4.00 ± 1.76^ab^	4.30 ± 1.89^bcdef^	4.90 ± 1.45^abcd^
S4	5.10 ± 2.38^abcd^	4.20 ± 2.10^ef^	6.00 ± 1.76^ab^	4.50 ± 2.07^abc^	4.10 ± 3.00^abc^	4.10 ± 2.28^abc^	3.90 ± 1.97^ab^	2.90 ± 1.73^f^	3.50 ± 1.43^de^
S5	5.90 ± 1.52^abc^	5.30 ± 1.57^cde^	4.80 ± 1.87^b^	4.30 ± 2.41^bc^	4.20 ± 3.16^abc^	4.20 ± 2.30^abc^	3.60 ± 1.78^ab^	3.70 ± 2.16^def^	4.20 ± 1.69^bcde^
S6	4.80 ± 1.14^bcd^	4.90 ± 1.37^def^	5.50 ± 1.08^b^	5.20 ± 0.79^ab^	4.60 ± 1.26^abc^	5.00 ± 0.94^ab^	5.40 ± 1.07^a^	6.40 ± 1.17^a^	5.30 ± 0.95^ab^
S7	5.40 ± 0.97^abcd^	5.50 ± 0.97^cde^	6.00 ± 1.56^ab^	5.70 ± 1.34^ab^	5.20 ± 1.40^ab^	5.10 ± 1.45^a^	5.20 ± 1.23^a^	5.00 ± 1.33^abcde^	5.10 ± 1.66^abc^

^a–f^Mean values with different superscripts in the same column are significantly different (*P* < 0.05).

**Table 3:** Correlation analysis between physicochemical characteristic values and sensory test on Korean *Makgeolli*.

	pH	Titratable acidity	Aminotype nitrogen	Soluble solid	Lightness	Redness	Yellowness	Turbidity	Color	Flavor	Sweetness	Sourness	Bitterness	Thickness	Cooling sensation	Balance
pH	NA	−0.910^**^	0.578	−0.038	0.033	0.498	0.141	0.35	−0.041	0.67	−0.353	−0.828^*^	−0.738	−0.703	−0.878^**^	−0.815^*^
Titratable acidity		NA	−0.509	0.134	−0.136	−0.476	−0.466	−0.559	−0.132	−0.622	0.368	0.913^**^	0.709	0.649	0.801^*^	0.716
Aminotype nitrogen			NA	0.716	0.351	0.933^**^	0.035	0.747	0.596	0.31	−0.101	−0.541	−0.477	−0.835^*^	−0.676	−0.463
Soluble solid				NA	0.344	0.63	−0.146	0.387	0.617	0.073	0.266	0.109	0.122	−0.359	−0.162	0.073
Lightness					NA	−0.186	−0.072	0.463	0.362	0.291	−0.068	−0.049	−0.078	−0.14	−0.039	−0.084
Redness						NA	0.092	0.879^*^	0.549	0.024	−0.099	−0.618	−0.491	−0.877^*^	−0.669	−0.405
Yellowness							NA	0.48	0.539	0.291	0.306	−0.302	0.13	0.064	0.011	0.219

Turbidity								NA	0.780^*^	0.105	−0.134	−0.603	−0.408	−0.649	−0.424	−0.242
Color									NA	0.11	0.481	−0.052	0.219	−0.201	0.057	0.337
Flavor										NA	0.252	−0.281	−0.075	−0.099	−0.368	−0.269
Sweetness											NA	0.648	0.861^*^	0.595	0.626	0.807^*^
Sourness												NA	0.898^**^	0.829^*^	0.891^**^	0.841^*^
Bitterness													NA	0.860^*^	0.887^**^	0.954^**^
Thickness														NA	0.904^**^	0.809^*^
Cooling sensation															NA	0.932^**^

^*^ *P* < 0.05; ^**^ *P* < 0.01. NA: not analysed.
